# A Review of Second- and Third-line Infertility Treatments and Supporting Evidence in Women with Polycystic Ovary Syndrome

**DOI:** 10.3390/medsci7070075

**Published:** 2019-06-26

**Authors:** Michael F. Costello, Rhonda M. Garad, Roger Hart, Hayden Homer, Louise Johnson, Cailin Jordan, Edgar Mocanu, Jie Qiao, Luk Rombauts, Helena J. Teede, Eszter Vanky, Christos A. Venetis, William L. Ledger

**Affiliations:** 1 University of New South Wales, High St. Kensington, Sydney, NSW 2052, Australia; c.venetis@unsw.edu.au (C.A.V.); w.ledger@unsw.edu.au (W.L.L.); 2 Monash Centre for Health Research and Implementation, Monash Public Health and Preventive Medicine, Monash University and Monash Health, Melbourne, VIC 3168, Australia; rhonda.garad@monash.edu (R.M.G.); helena.teede@monash.edu (H.J.T.); 3 Division of Obstetrics and Gynaecology, The University of Western Australia, University of Western Australia, Crawley, WA 6008, Australia; roger.hart@uwa.edu.au; 4 Christopher Chen Oocyte Biology Research Laboratory, UQ Centre for Clinical Research, The University of Queensland, Brisbane, QLD 4029, Australia; h.homer@uq.edu.au; 5 Victorian Assisted Reproductive Treatment Authority, Melbourne, VIC 3000, Australia; LJohnson@varta.org.au; 6 Genea Hollywood Fertility, Wembley 6014, Australia; Cailin.Jordan@genea.com.au; 7 Royal College of Surgeons, Rotunda Hospital, D02 YN77 Dublin, Ireland; emocanu@rcsi.ie; 8 Department of Obstetrics and Gynaecology, Medical Center for Human Reproduction, Peking University Third Hospital, Beijing 100191, China; jie.qiao@263.net; 9 Department of Obstetrics and Gynaecology, Monash University, Melbourne 3168, Australia; luk.rombauts@monash.edu; 10 National Health and Medical Research Council Centre for Research Excellence in PCOS, Monash University, Melbourne 3168, VIC, Australia; 11 National Health and Medical Research Council Centre for Research Excellence in PCOS, University of Adelaide, Adelaide 5006, SA, Australia; 12 University of New South Wales, Sydney 2033, NSW, Australia; 13 Department of Clinical and Molecular Medicine, Norwegian University of Science and Technology, 1517 Trondheim, Norway; eszter.vanky@ntnu.no

**Keywords:** polycystic ovary syndrome, infertility, surgery, assisted reproductive technology, IVF, IVM

## Abstract

In clomiphene-citrate-resistant anovulatory women with polycystic ovary syndrome (PCOS) and no other infertility factors, either metformin combined with clomiphene citrate or gonadotrophins could be used as a second-line pharmacological therapy, although gonadotrophins are more effective. Gonadotrophins could also be used as a second-line pharmacological therapy in anovulatory women with PCOS and clomiphene-citrate-failure. Laparoscopic ovarian surgery can also be used as a second-line therapy for ovulation induction in anovulatory women with clomiphene-citrate-resistant PCOS and no other infertility factors. The usefulness of letrozole as a second-line pharmacological treatment for ovulation induction in clomiphene-citrate-resistant women with PCOS requires further research. In terms of improving fertility, both pharmacological anti-obesity agents and bariatric surgery should be considered an experimental therapy in anovulatory women with PCOS and no other infertility factors. Where first- or second-line ovulation induction therapies have failed, in vitro fertilization (IVF)/ intracytoplasmic sperm injection (ICSI) could be offered as a third-line therapy in women with PCOS in the absence of an absolute indication for IVF/ICSI. For women with PCOS undergoing IVF/ICSI treatment, the gonadotropin-releasing hormone (GnRH) antagonist protocol is preferred and an elective frozen embryo transfer strategy could be considered. In assisted conception units with sufficient expertise, in-vitro maturation (IVM) of oocytes could be offered to women with PCOS.

## 1. Introduction

Polycystic ovary syndrome (PCOS), with a prevalence of between 8% and 13%, depending on the population studied and definitions used, is the most common endocrinopathy affecting reproductive-aged women. PCOS is a complex condition with reproductive, metabolic and psychological features [1]. A diagnosis of PCOS is based on the three characteristics of anovulation and/or oligo-ovulation, clinical or biochemical hyperandrogenism and polycystic ovaries [2]. 

Infertility is a prevalent presenting feature of PCOS with 75% of these women suffering infertility due to anovulation, making PCOS the most common cause of anovulatory infertility [3]. The treatment of infertility for women with PCOS includes lifestyle changes (diet, exercise and behavioural strategies), pharmacological therapies (oral agents such as clomiphene citrate, letrozole or metformin or injectable agents such as gonadotrophins), surgical therapy (laparoscopic ovarian surgery) or in vitro fertilization (IVF) [4]. A promising alternative to conventional IVF that has been proposed is in vitro maturation (IVM) [5]. First-line pharmacological treatment for infertile women with PCOS may include letrozole, clomiphene citrate, metformin, clomiphene citrate combined with metformin and gonadotrophins, of which letrozole is considered the first-line therapy [6,7].

The aim of this narrative review is to provide a summary and brief update of the best available and most current research evidence that evaluates the second- and third-line treatment of PCOS-related infertility and which has informed the recommendations in the assessment and treatment of infertility section of the International Evidence-Based Guideline on PCOS [6,7]. The methodology for the literature search and selection of studies for this narrative review are described in the guideline and the guideline technical report. The Grading of Recommendations, Assessment, Development, and Evaluation (GRADE) framework was applied across the quality of evidence (QOE) [8]. 

## 2. Letrozole

### 2.1. Background

Letrozole, an aromatase inhibitor, was first proposed as an ovulation-inducing drug in anovulatory PCOS women in 2001 [9]. Letrozole is usually administered on days 3–7 of the menstrual cycle at doses of 2.5–7.5 mg per day in 2.5 mg increments [10]. 

### 2.2. Evidence

The recently published international evidence-based guideline on PCOS identified a single published randomised controlled trial (RCT) comparing letrozole versus placebo or no treatment with timed intercourse in PCOS [11]. This RCT showed insufficient evidence for a difference between letrozole and placebo in live birth (1/18 women; 6% vs. 0/18 women; 0% respectively; *p* = 0.324) and clinical pregnancy (1/18 women; 6% vs. 0/18 women; 0% respectively; *p* = 0.324) rates but a higher ovulation rate (6/18 women; 33% vs. 0/18 women; 0% respectively; *p* = 0.006) per woman with letrozole in clomiphene-citrate-resistant PCOS women. There were no cases of miscarriage or multiple pregnancies in the RCT.

### 2.3. Summary

The use of letrozole as a second-line pharmacological treatment for ovulation induction in anovulatory women with clomiphene-citrate-resistant PCOS and no other infertility factors has been shown to improve ovulation rates. Further large, adequately powered, well conducted and reported RCTs are required comparing letrozole versus placebo or no treatment in anovulatory women with PCOS with clomiphene citrate resistance or failure to properly evaluate letrozole as a second-line treatment agent.

## 3. Metformin Combined with Clomiphene Citrate

### 3.1. Background

Metformin, an insulin-sensitizing drug, was first reported as a treatment for PCOS in 1994 where it was found to facilitate normal menses and pregnancy at doses of 1500 mg daily [12]. Clomiphene citrate, a selective estrogen receptor modulator, was first reported to induce ovulation in 1961 [13] and has been used as a first-line medical ovulation induction agent since 1967 [10]. Clomiphene citrate is administered for five days beginning on any menstrual cycle day from 2 to 5, starting with 50 mg/day and increasing to 150 mg/day if anovulatory. If ovulation cannot be achieved at doses of 150 mg/day, the patient is deemed to have clomiphene citrate resistance. If pregnancy cannot be achieved after six ovulatory cycles, then the patient is deemed to have clomiphene-citrate-failure [14]. The first study evaluating metformin combined with clomiphene citrate was an RCT published in 1998 [15,16].

### 3.2. Evidence

The most recent systematic reviews and pairwise meta-analyses of RCTs that have compared the combination of metformin plus clomiphene citrate versus clomiphene citrate alone in women with PCOS who were clomiphene-citrate-resistant showed that metformin combined with clomiphene citrate had a higher live birth, pregnancy and ovulation rate compared with clomiphene citrate alone [17,18]. Therefore, the addition of metformin to clomiphene citrate is more beneficial in clomiphene-citrate-resistant women with PCOS than persisting with further cycles of clomiphene citrate alone. 

This Cochrane systematic review and pairwise meta-analysis of RCTs also compared metformin combined with clomiphene citrate versus letrozole [19] and located a single RCT of 250 clomiphene-citrate-resistant women with PCOS which showed no evidence of a difference between the two treatment arms in terms of live-birth, clinical pregnancy, multiple pregnancy and miscarriage rates; however, the results were inconclusive due to the wide 95% confidence intervals [20].

### 3.3. Summary

Metformin combined with clomiphene citrate could be used as second-line pharmacological therapy in anovulatory women with PCOS and no other infertility factors, as it is superior to clomiphene citrate alone in clomiphene-citrate-resistant women. 

## 4. Gonadotrophins

### 4.1. Background

Ovulation induction with gonadotrophins began in the 1960s and there is a large body of observational evidence supporting the use of gonadotrophin ovulation induction in clomiphene citrate resistant or clomiphene-citrate-failure PCOS women, with the use of the low dose step-up protocol with typical starting doses of follicle-stimulating hormone (FSH) 50–75 IU being recommended in order to reduce the risk of multiple pregnancy and ovarian hyperstimulation syndrome [21]. 

### 4.2. Evidence

The evidence is applicable to the both urinary and recombinant gonadotrophins including human menopausal (urinary) gonadotrophins (HMG), highly-purified urinary FSH (HP-uFSH) and recombinant FSH (rFSH). 

#### 4.2.1. Gonadotrophins versus Placebo/No Treatment

The recently published international evidence-based guideline on PCOS [6] and the recently published systematic review and network meta-analysis of RCTs on ovulation induction in World Health Organization (WHO) Group 2 (including PCOS) anovulatory women [22] did not find any published RCTs directly comparing gonadotrophins versus placebo/no treatment. However, the latter systematic review demonstrated higher pregnancy (odds ratio (OR): 5.71; 95% confidence interval (CI) 2.18–15.00) and ovulation (OR 21.86; 95% CI 3.06–156.02) rates with gonadotrophins but insufficient evidence to determine if there was a difference in live birth (OR 5.24; 95% CI 0.57–48.09), miscarriage (OR 1.91; 95% CI 0.30–12.22) or multiple pregnancy (OR 0.54; 95% CI 0.10–291.45) rates per woman between the two interventions upon network meta-analysis. 

#### 4.2.2. Gonadotrophins versus Other Ovulation Induction Therapies

A recently published multi-centre Dutch RCT compared gonadotrophins with timed intercourse or intra-uterine insemination (IUI) versus clomiphene citrate with timed intercourse or IUI over six cycles in 666 infertile WHO Group 2 anovulatory women with clomiphene-citrate-failure (ovulatory for six clomiphene citrate 150 mg maximum dose ovulation induction cycles but not conceived) and found a higher live-birth rate (52% vs. 41%; relative risk (RR) 1.24; 95% CI 1.05–1.46; *p* = 0.0124) and reduced time to conception leading to live-birth (5.0 vs. 5.5 months; *p* = 0.028) favouring gonadotrophins and the addition of IUI did not increase live-births compared with timed intercourse (49% vs. 43%; RR 1.14; 95% CI 0.97–1.35; *p* = 0.1152). There was no evidence of a difference in multiple pregnancy rates (2% vs. 2%; RR = 0.89; 95% CI 0.33–2.4; *p* = 0.826) [23].

However, gonadotrophins are superior to metformin combined with clomiphene citrate for ovulation induction in women with PCOS who are clomiphene-citrate-resistant in terms of live-birth/ongoing pregnancy (OR 0.33; 95% CI 0.13–0.85; 2 RCTs; 170 women), clinical pregnancy (OR 0.45; 95% CI 0.27–0.75; 3 RCTs; 323 women) and ovulation rates (OR 0.25; 95% CI 0.15–0.41; 3 RCTs; 323 women; *I*^2^ 85%), as evidenced by a systematic review and pairwise meta-analysis of RCTs [24]. 

A Cochrane systematic review and pairwise meta-analysis of RCTs compared gonadotrophins versus letrozole [19] and located a single RCT of 140 clomiphene-citrate-resistant PCOS women which showed no evidence of a difference between the two therapies in terms of clinical pregnancy and multiple pregnancy rates per woman; however, the results were inconclusive due to the wide 95% confidence intervals [25]. A network meta-analysis comparing gonadotrophins versus letrozole also found no evidence of a difference in reproductive outcomes including the primary outcome of pregnancy rate per randomized woman for all WHO Group 2 (including PCOS) anovulatory women (OR 1.07; 95% CI 0.52–2.21), with the inconclusive results explained by the wide 95% confidence intervals [22]. 

The co-administration of metformin to gonadotrophins for ovulation induction with timed intercourse or intra-uterine insemination has been demonstrated to improve live-birth (OR 2.31; 95% CI 1.23–4.34; 2 RCTs; 180 women), ongoing pregnancy (OR 2.46; 95% CI 1.36–4.46; 4 RCTs; 232 women) and clinical pregnancy (OR 2.51; 95% CI 1.46–4.31; 5 RCTs; 264 women) rates with no evidence of a difference in miscarriage (OR 0.62 favouring gonadotrophins; 95% CI 0.19–2.01; 3 RCTs; 84 women), multiple pregnancy (OR 0.55 favouring gonadotrophins; 95% CI 0.15–1.95; 4 RCTs; 232 women), or ovarian hyperstimulation syndrome (OHSS) (OR 0.32 favouring gonadotrophins; 95% CI 0.01–8.23; 2 RCTs; 180 women) rates in clomiphene-citrate-resistant PCOS women with anovulatory infertility in a Cochrane systematic review and pair-wise meta-analysis of five RCTs [26]. The dose of metformin varied between 1500 and 1700 mg daily. The metformin treatment commenced between one (four RCTs) and six (one RCT) months prior to gonadotrophins and stopped with either human chorionic gonadotropin (hCG) trigger (three RCTs) or pregnancy test (two RCTs). 

### 4.3. Summary

Gonadotrophins could be used as a second-line pharmacological therapy in cases where first-line pharmacological therapy has failed in anovulatory women with PCOS and no other infertility factors, as they are more effective than placebo/no treatment (all WHO Group 2 women), clomiphene citrate alone (clomiphene-citrate-failure women), and metformin combined with clomiphene citrate (clomiphene-citrate-resistant women). The addition of metformin to gonadotrophins could be considered in clomiphene-citrate-resistant PCOS women as it is superior to gonadotrophins alone. 

## 5. Laparoscopic Ovarian Surgery 

### 5.1. Background

Laparoscopic ovarian surgery (LOS) was first described in women with PCOS by Gjonnaess in 1984 [27]. Both laparoscopic ovarian cautery (unipolar or bipolar) and laser vaporisation using carbon dioxide (CO2), argon or neodymium-doped yttrium aluminium garnet (Nd:YAG; Nd: Y3Al5O12) crystal lasers have been used (either bilaterally or unilaterally) with no standard number/depth of perforations or dose/duration of energy sources to the ovary for optimal results [28,29]. 

### 5.2. Evidence

Laparoscopic ovarian surgery is a second-line therapy in clomiphene-citrate-resistant women with PCOS with no evidence of a difference compared to 3–6 treatment cycles of gonadotrophin ovulation induction in terms of the live birth rate per woman (OR 0.97 favouring LOS; 95% CI 0.59–1.57; 3 RCTs; 318 women; Mod QOE), pregnancy rate per woman (OR 1.01 favouring gonadotrophins; 95% CI 0.72–1.42; 8 RCTs; 607; women; Mod QOE), ovulation rate per woman (OR 0.66 favouring LOS; 95% CI 0.21–2.07; 1 RCT; 50 women; Mod QOE), miscarriage rate per pregnancy (OR 0.73 favouring LOS; 95% CI 0.40–1.33; 7 RCTs; 441 women; Mod QOE) and OHSS rate per woman (OR 0.08 favouring LOS; 95% CI 0.00–1.61; 3 RCTs; 251 women; Mod QOE) but with a lower risk of multiple pregnancy per pregnancy (OR 0.13; 95% CI 0.03–0.52; 5 RCTs; 166 women; High QOE) and lower cost [29].

#### Laparoscopic Ovarian Surgery versus Other Ovulation Induction Therapies

There are two RCTs that compare LOS versus metformin in clomiphene-citrate-resistant women with PCOS which show conflicting findings [30,31]. The RCT by Hamed et al. demonstrated a higher pregnancy rate per patient (38.2% (21/55) vs. 20.0% (11/55); *p* < 0.03) and a higher ovulation rate per cycle (50.8% (131/258) vs. 33.5% (94/281); *p* < 0.001) with LOS with no evidence of a difference in THE miscarriage rate per pregnancy whilst the RCT by Palomba and colleagues reported a lower live-birth rate per pregnancy (64.5% (20/31) vs. 82.1% (32/39); *p* < 0.05), lower pregnancy rate per cycle (13.4% (31/231) vs. 18.6% (39/210); *p* < 0.05) and higher miscarriage rate per pregnancy (29% (9/31) vs. 15.4% (6/39); *p* < 0.05) with LOS. 

Laparoscopic ovary surgery versus clomiphene citrate in clomiphene-citrate-failure women with PCOS has been compared in a single RCT which demonstrated no evidence of a difference in live-birth or pregnancy rates per patient between the two interventions and increased miscarriage rates per pregnancy with LOS [32].

A Cochrane systematic review and meta-analysis has compared LOS versus metformin combined with clomiphene citrate in clomiphene-citrate-resistant women with PCOS and showed a decrease in the live-birth rate with LOS (OR 0.44; 95% CI 0.24–0.82; 2 RCTs; 159 women; *I*^2^ 78%). There was insufficient evidence to determine if there was a difference in pregnancy, ovulation, miscarriage and multiple pregnancy rates [29].

LOS has been compared to letrozole for ovulation induction in a recently published Cochrane systematic review and pair-wise meta-analysis [19]. There was no evidence of a difference in live-birth (OR 1.38 favouring letrozole; 95% CI 0.95–2.02; 3 RCTs; 548 women), clinical pregnancy (OR 1.30 favouring letrozole; 95% CI 0.93–1.83; 4 RCTs; 628 women), miscarriage (OR 0.69; 95% CI 0.29–1.63; 4 RCTs; 628 women) and multiple pregnancy (OR 3.0; 95% CI 0.12–74.90; 3 RCTs; 548 women) rates per patient randomized in clomiphene-citrate-resistant women with PCOS. 

A single RCT has evaluated LOS versus letrozole combined with metformin in clomiphene-citrate-resistant women with PCOS and found no evidence of a difference in pregnancy rate per patient, ovulation rate per patient and miscarriage rate per pregnancy [33]. 

### 5.3. Summary

Laparoscopic ovary surgery can be used as a second-line therapy for ovulation induction in anovulatory women with clomiphene-citrate-resistant PCOS and no other infertility factors. Metformin cannot be advocated as a second-line therapy given that the current evidence does not consistently support it’s superiority over that of LOS in terms of the reproductive outcome. 

## 6. Anti-Obesity Agents

### 6.1. Background

Phentermine, a sympathomimetic amine which decreases appetite, has been approved for short-term weight loss since 1959 and is usually prescribed at doses of 15–40 mg daily. Orlistat, a pancreatic lipase inhibitor that decreases the absorption of ingested fat, was initially approved by the Food and Drug Administration (FDA) in 1999 and is typically taken at doses of 120 mg with each main meal up to three times daily [34]. 

### 6.2. Evidence

No published RCTs on pharmacological anti-obesity agents (Orlistat, Phenteramine) in PCOS were identified by the recently published international evidence-based guideline on PCOS [6]. Therefore, in the absence of such evidence, the guideline document reported a clinical consensus recommendation stating that, for the purpose of improving fertility in women with PCOS, pharmacological anti-obesity agents should be considered as an experimental therapy, with risk to benefit ratios currently too uncertain to advocate this as fertility therapy.

### 6.3. Summary

Pharmacological anti-obesity agents, for the purpose of improving fertility, should be considered an experimental therapy in anovulatory women with PCOS and no other infertility factors. 

## 7. Bariatric Surgery

### 7.1. Background

Bariatric surgery is usually indicated for patients with a body mass index (BMI) of ≥40 kg/m^2^ or for patients with a BMI of ≥35 kg/m^2^ with at least one obesity-related comorbidity who have failed conservative treatment. Bariatric surgery contributes to weight loss by either restriction (limiting the amount of food the stomach can hold, which limits the number of calories consumed) or malabsorption (shorten or bypass part of the small intestine, which reduces the number of calories absorbed by the body). Common bariatric surgeries include vertical sleeve gastrectomy (VSG), laparoscopic adjustable gastric banding, Roux-en-Y gastric bypass, duodenal switch with biliopancreatic diversion and several minimally invasive devices including intra-gastric balloons [34].

### 7.2. Evidence

No published RCTs on bariatric surgery in PCOS were identified by the recently published international evidence-based guideline on PCOS [6]. Therefore, in the absence of such evidence, the guideline document reported a clinical consensus recommendation stating that, in women with PCOS, for the purpose of having a healthy baby, bariatric surgery should be considered as an experimental therapy with risk-to-benefit ratios currently too uncertain to advocate this as a fertility therapy.

### 7.3. Summary

Bariatric surgery, for the purpose of improving fertility, should be considered an experimental therapy in anovulatory women with PCOS and no other infertility factors. 

## 8. In Vitro Fertilization/Intracytoplasmic Sperm Injection Treatment

### 8.1. Background

The world’s first IVF child, Louise Brown, was born in the United Kingdom on 25 July 1978 [35]. 

### 8.2. Evidence

The recently published international evidence-based guideline on PCOS did not identify any published RCTs comparing IVF treatment with ovulation induction treatments or placebo/no treatment in PCOS [6]. In the absence of such evidence, this guideline document reported a clinical consensus recommendation stating that, in the absence of an absolute indication for IVF/ICSI, women with PCOS and anovulatory infertility could be offered IVF/ICSI as third-line therapy where first- or second-line ovulation induction therapies have failed. 

The gonadotropin-releasing hormone (GnRH) antagonist protocol has been compared with the GnRH agonist long protocol in women with PCOS undergoing IVF or ICSI in a recently published systematic review and meta-analysis [36]. There was insufficient evidence for a difference in live-birth (RR 0.90; 95% CI 0.69–1.19; 3 RCTs; 363 women), ongoing pregnancy (RR 0.97 favouring GnRH antagonist protocol; 95% CI 0.84–1.11; 9 RCTs; 1294 women) and clinical pregnancy (RR 1.01; 95% CI 0.86–1.19; 10 RCTs; 1086 women) rates per woman randomized and also, the number of oocytes retrieved (MD 0.40; 95% CI −0.97 to 1.77; 7 RCTs; 965 women) between the two protocols. However, there was a reduced risk of ovarian hyperstimulation syndrome with the GnRH antagonist protocol (RR 0.53; 95% CI 0.30–0.95; 9 RCTs; 1294 women). 

There has been a single RCT comparing Human Menopausal Gonadotrophin versus recombinant FSH in women with PCOS undergoing IVF using a GnRH agonist long protocol, demonstrating no evidence of a difference in live-birth (23.1% vs. 35.7% respectively; *p* = 0.27) or clinical pregnancy (23.1% vs. 40.5% respectively; *p* = 0.14) rates per patient, total dose of gonadotrophins (1716 IU vs. 1429 IU respectively; *p* = 0.57) and ovarian hyperstimulation syndrome (0% vs. 11.9% respectively; *p* = 0.14). However, Human Menopausal Gonadotrophin had a longer duration of ovarian stimulation (+1.1 days; *p* = 0.025), lower maximum serum E2 level (−899 pg/mL; *p* = 0.02) and lower number of eggs collected (−4.06 eggs; *p* = 0.002) compared to recombinant FSH [37]. 

A Cochrane systematic review and meta-analysis has evaluated the effect of adjuvant metformin therapy in conjunction with IVF or ICSI in women with PCOS [38,39]. This review found insufficient evidence for a difference in the live-birth rate (OR 1.39 favouring metformin; 95% CI 0.81–2.40; 5 RCTs; 551 women; low QOE) or miscarriage rate (OR 0.76 favouring metformin; 95% CI 0.43–1.37; 6 RCTs; 527 women; moderate QOE) per woman with the use of metformin therapy before and/or during FSH ovarian stimulation in a GnRH agonist long protocol, whilst adjunct metformin resulted in a higher clinical pregnancy rate (OR 1.52; 95% CI 1.07–2.15; 8 RCTs; 775 women; moderate QOE), reduced OHSS rate (OR 0.29; 95% CI 0.18–0.49; 8 RCTs; 798 women; moderate QOE) and increased side-effects (gastro-intestinal) (OR 4.49; 95% CI 1.88–10.72; 4 RCTs; 431 women; low QOE) per woman. The review located a single RCT evaluating the effect of adjuvant metformin therapy in a GnRH antagonist protocol which, of the above outcomes, only reported on OHSS and demonstrated insufficient evidence for a difference in the OHSS rate per woman (OR 0.30 favouring metformin; 95% CI 0.03–3.15; 1 RCT; 40 women) [40]. In this Cochrane review on the effect of adjuvant metformin therapy in conjunction with IVF or ICSI in women with PCOS, the dose of metformin ranged from 1000 to 2550 mg daily, with the metformin commencing from 16 weeks prior to FSH ovarian stimulation to the day of commencing FSH ovarian stimulation, and the last dose of metformin either on day of hCG trigger, egg collection, embryo transfer, pregnancy test or 12 weeks gestation [38]. 

A multi-centre (*n* = 14) Chinese RCT has compared fresh versus elective frozen embryo transfer (FET) in 1508 infertile women with PCOS undergoing their first IVF cycle using the GnRH antagonist protocol with urinary hCG trigger [41]. The intervention of embryo freezing on day three followed by later hormone replacement frozen embryo transfer (HRT FET) using oral estradiol valerate plus intra-muscular progesterone until 10 weeks of pregnancy was compared with a control group who underwent fresh embryo transfer (ET) on day three with a luteal phase support consisting of intra-muscular progesterone daily from the day of egg collection until 10 weeks of pregnancy. The FET group had a higher live-birth rate per embryo transfer (49.3% vs. 42.0%; risk difference (RD) = 7.3%; 95% CI 2.3–12.4; *p* = 0.004), singleton birth weight (3511 g vs. 3349 g; *p* = 0.005) and risk of pre-eclampsia (4.4% vs. 1.4%; RD = 3.0%; 95% CI 0.8–5.2: *p* = 0.009) with a lower clinical pregnancy loss rate (14.6% vs. 25%; RD = −10.4%; 95% CI −15.7 to −5.1; *p* < 0.001) and risk of moderate or severe ovarian hyperstimulation syndrome (1.3% vs. 7.1%; RD = −5.7%; 95% CI −7.7 to −3.7; *p* < 0.001). There was no evidence of a difference between FET and fresh ET groups in terms of biochemical (66% vs. 64.6% favouring FET; RD = 1.4%; 95% CI −3.4 to 6.2; *p* = 0.57), clinical (58.7% vs. 56.2% favouring FET; RD = 2.5%; 95% CI −2.4 to 7.5; *p* = 0.32) and ongoing pregnancy (52.7% vs. 48.8% favouring FET; RD = 3.9%; 95% CI −1.1% to 8.9%; *p* = 0.13) rates.

### 8.3. Summary

In vitro fertilization/ICSI could be offered as third-line therapy in women with PCOS, where first- or second-line ovulation induction therapies have failed, in the absence of an absolute indication for IVF/ICSI. In women with PCOS undergoing IVF/ICSI treatment, the GnRH antagonist protocol is preferred over the GnRH agonist long protocol, either Human Menopausal Gonadotrophin or recombinant FSH can be used and elective frozen embryo transfer strategy could be considered. If using the GnRH agonist long protocol, adjunct metformin therapy could be used to improve clinical outcome.

## 9. In Vitro Maturation Treatment

### 9.1. Background

Where IVF is indicated in PCOS, OHSS risks are increased with gonadotrophin stimulation. IVM of oocytes limits or omits ovarian stimulation prior to oocyte retrieval, with maturation of oocytes post retrieval, avoiding OHSS risk [5].

### 9.2. Evidence

No published RCTs comparing IVM treatment with IVF/ICSI or ovulation induction treatments or placebo/no treatment in women with PCOS were identified by the recently published international evidence-based guideline [6]. Therefore, in the absence of such evidence, the guideline document reported a clinical consensus recommendation stating that, for women with PCOS, IVM could be offered in units with sufficient expertise to achieve pregnancy and live-birth rates approaching those of standard IVF/ICSI treatment without the risk of OHSS, where an embryo is generated, then vitrified and thawed and transferred in a subsequent cycle.

### 9.3. Summary

In vitro maturation, where an embryo is generated, then vitrified and thawed and transferred in a subsequent cycle, could be offered to women with PCOS in assisted reproduction units with sufficient expertise. 

## 10. Conclusions

The evidence and summaries for second- and third-line ovulation induction in this review are applicable to women with PCOS who have anovulatory infertility and no other infertility factors. The usefulness of letrozole as a second-line pharmacological treatment for ovulation induction in clomiphene-citrate-resistant PCOS requires further study. Metformin combined with clomiphene citrate could be used as second-line pharmacological therapy in clomiphene-citrate-resistant women, although gonadotrophins are more effective. Gonadotrophins could also be used as second-line pharmacological therapy in clomiphene-citrate-failure women. Laparoscopic ovarian surgery can also be used as second-line ovulation induction therapy in women with clomiphene citrate resistance. Both pharmacological anti-obesity agents and bariatric surgery, with the aim of improving fertility, should be considered as an experimental therapy. In the absence of an absolute indication for IVF/ICSI, IVF/ICSI could be offered as third-line therapy in women with PCOS, where first- or second-line ovulation induction therapies have failed. In women with PCOS undergoing IVF/ICSI treatment, the GnRH antagonist protocol is preferred and an elective frozen embryo transfer strategy could be considered. IVM could be offered to women with PCOS in assisted reproduction units with sufficient expertise.
